# Overexpression of xanthine dehydrogenase extends lifespan in *C. elegans*

**DOI:** 10.17912/micropub.biology.002118

**Published:** 2026-07-04

**Authors:** Asuka Chino, Ayaka Sugiyama, Hayao Ohno

**Affiliations:** 1 Division of Material and Biological Sciences, Graduate School of Science, Japan Women's University, Tokyo, Japan; 2 Department of Chemical and Biological Sciences, Faculty of Science, Japan Women's University, Tokyo, Japan

## Abstract

Uric acid is known to act as an antioxidant, and one hypothesis posits that certain primates, including humans, increased their uric acid levels during evolution to extend lifespan. To test whether genetically altering the activity of endogenous uric acid synthesis affects organismal lifespan, we generated transgenic *Caenorhabditis elegans* overexpressing the xanthine dehydrogenase XDH-1 , an enzyme involved in uric acid production. These transgenic animals displayed a 9–15% increase in lifespan. They also exhibited enhanced resistance to the oxidative stress–inducing agent paraquat, implying that the lifespan extension might be linked to the antioxidant effects of uric acid.

**Figure 1. Overexpression of xdh-1 in a WT background results in lifespan extension and enhanced resistance to paraquat:** ( **A** ) Lifespans of WT animals and transgenic animals expressing&nbsp; *xdh-1* &nbsp;driven by the&nbsp; *eft-3* &nbsp;promoter ( *Ex* [ *eft-3 p:: xdh-1* ]). Number of animals analyzed and P values are shown in Table 1A, Experiment 1. ( **B** ) Lifespans of WT and *xdh-1 ( ok3234 )* animals. Number of animals analyzed and P value are shown in Table 1B, Experiment 1. ( **C** ) Pharyngeal pumping rate of&nbsp;WT and *Ex* [ *eft-3 p:: xdh-1* ] ( CAT33 ). Bars represent mean ± SEM. Number of animals analyzed: *n* = 16. *P* = 0.8329 (two-tailed *t* test). n.s., not significant. ( **D** , **E** ) Fraction of WT and *Ex* [ *eft-3 p:: xdh-1* ] animals grown to L4 or adult stages in the presence of 0.2 mM (D) and 0.4 mM (E) paraquat. Bars represent mean ± SEM. *n* = 3 assays. The total numbers of animals analyzed in all assays are shown in parentheses. Two-tailed *t* test. n.s., not significant. **Table 1. Results of lifespan analysis:** ( **A** , **B** ) LS, lifespan. Statistical analyses were conducted using Log-rank (Mantel-Cox) test.

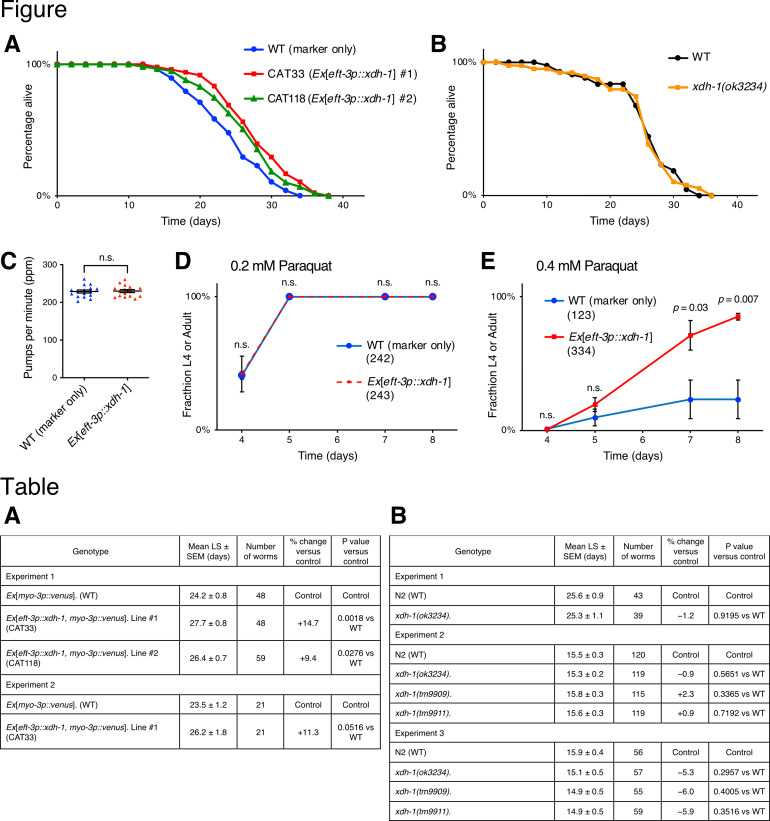



## Description

Uric acid is generated through the oxidation of xanthine catalyzed by xanthine dehydrogenase (XDH) or xanthine oxidase (XO) (Chung et al., 1997; Bortolotti, 2021). It constitutes the terminal metabolite of purine nucleotide catabolism in humans and in many other species it serves as a primary nitrogenous excretion product. Uric acid exhibits potent antioxidant activity and has been implicated in influencing organismal lifespan (Ames et al., 1981; Glantzounis et al., 2005). A positive correlation has been reported between primate species' maximum lifespan and plasma uric acid concentration (Cutler, 1991), and it has been proposed that evolutionary alterations in uric acid metabolism—such as the loss of uricase—have contributed to lifespan changes in species including humans (Álvarez-Lario & Macarrón-Vicente, 2010).

It has been reported that addition of uric acid to the culture medium extends the lifespan of *Caenorhabditis elegans* (Wan et al., 2020). However, this effect may be indirect: uric acid could adversely affect the bacterial food source, producing calorie-restriction–like conditions (Lakowski & Hekimi, 1998; Lee et al., 2006) or altering bacterial growth activity, which can affect worm lifespan (Fukushima et al., 2025; Garsin et al., 2001). The *C. elegans* gene *xdh-1* encodes XDH-1 , the worm homolog of xanthine dehydrogenase (Yoshina et al., 2022). XDH-1 functions in AIN and AVJ neurons to regulate cold tolerance (Takagaki et al., 2020), and loss of *xdh-1* activity promotes formation of xanthine stones (Snoozy et al., 2025).

In the present study, we generated a transgenic *C. elegans* line that overexpresses *xdh-1* and measured lifespan to address whether altering the activity of the endogenous uric-acid synthesis pathway changes organismal lifespan. The *xdh-1* cDNA was placed under the *eft-3* (also known as *eef-1A.1* ) promoter ( *eft-3 p* ) to drive strong, ubiquitous expression and introduced into wild-type animals; the resulting transgenic line was designated CAT33 . Although *eft-3 p* has been employed in previous investigations of lifespan (e.g., Tuckowski et al., 2025; Morphis et al., 2022), its introduction per se has not been reported to affect lifespan. In addition, the *xdh-1* cDNA has been confirmed to be functionally active (Takagaki et al., 2020). CAT33 exhibited an approximately 15% increase in lifespan compared with wild type ( Fig. 1A and Table 1A). To confirm reproducibility, lifespan was measured again one month later; this experiment again showed an extension of about 11% (Table 1A). An independently obtained transgenic line carrying the same construct ( CAT118 ) also showed lifespan extension (Table 1A). By contrast, three *xdh-1* loss-of-function mutants, *xdh-1 ( ok3234 )* , *xdh-1 ( tm9909 )* , and *xdh-1 ( tm9911 )* , showed no change in lifespan relative to wild-type ( N2 ) animals, suggesting that overexpression of *xdh-1* is sufficient for lifespan extension but *xdh-1* is not necessary for normal lifespan ( Fig. 1B and Table 1B). Together, these results indicate that appropriately altering activity in the uric acid synthesis pathway can potentially extend lifespan.

The pharyngeal pumping rate of CAT33 did not differ from that of wild-type animals ( Fig. 1C ), suggesting that dietary restriction is unlikely to account for the observed lifespan extension. To assess whether CAT33 exhibits increased oxidative stress resistance, we cultured worms on media containing the oxidative stressor paraquat; in the presence of 0.4 mM paraquat, CAT33 showed enhanced resistance ( Fig. 1D and 1E). It is conceivable that uric acid, elevated by XDH-1 overexpression, could act as an antioxidant and thereby extend lifespan. However, whether XDH-1 overexpression actually increases uric acid levels, whether any increase in uric acid is causally responsible for lifespan extension, which tissues XDH-1 acts in to modulate lifespan, and whether optimizing the level or site of XDH-1 expression could further extend longevity remain open questions for future study.

## Methods

For pDEST- *xdh-1* , the&nbsp;SalI-KpnI fragment from pNTN036 (a gift from A. Kuhara), which contains the *xdh-1* cDNA (Takagaki et al., 2020), was subcloned into the XhoI-KpnI site of pPD-DEST2-exman (a gift from H. Kunitomo). The PCR-amplified *eft-3* promoter (2,852 bp) was cloned into pDONR201 through BP reaction (site-specific recombination) to create pENTR- *eft-3 p* . The expression constructs of pG- *eft-3 p:: xdh-1* was created by LR reaction between pENTR *- eft-3 p* and pDEST- *xdh-1* . Details of the system are available at the following web site:

http://molecular-ethology.biochem.s.u-tokyo.ac.jp/Gateway/Gateway_overview1.html

Germ-line transformations were performed using standard microinjection methods. For the CAT33 strain, pG- *eft-3 p:: xdh-1* was injected at 20 ng/µL along with the co-injection marker pG- *myo-3p::venus* (15 ng/µL) and the carrier DNA plasmid pPD49.26 (65 ng/µL).

Lifespan assays and pharyngeal pumping assays were performed as described previously (Ohno et al., 2017), with exception that the lifespan assay plates were incubated at 20°C. Note: For preparation of NGM plates used in lifespan assays, N1000 Nematode Growth Medium (USBiological, Swampscott, MA, USA) was used in Table 1A, Experiments 1 and 2 and Table 1B, Experiment 1, whereas HIPOLYPEPTON SHIOTANI (SHIOTANI M.S., Hyogo, Japan) was used in Table 1B, Experiments 2 and 3. The latter formulation supports more robust microbial growth; this difference may have contributed to the shortened mean lifespan observed in Table 1B, Experiments 2 and 3.

For paraquat assays, nematode growth medium (NGM) plates containing paraquat (0.2 or 0.4 mM) were prepared by diluting a 1 M paraquat stock solution into molten NGM prior to dispensing. Plates were seeded with *E. coli* HB101 . Gravid adults were placed on the plates and allowed to lay eggs overnight; adults were removed the following day (day 1). The proportion of animals that had reached the L4 larval stage was scored on days 4, 5, 7, and 8.

Statistic analyses were performed using Prism v.10 (GraphPad software, San Diego, CA).&nbsp;

## Reagents

Strains used in this study:

**Table:** 

N2	*Caenorhabditis elegans* wild isolate.
RB2379	*xdh-1 ( ok3234 )* IV. ^(a)^
FX33446	*xdh-1 ( tm9909 )* IV. ^(b)^
FX33448	*xdh-1 ( tm9911 )* IV. ^(b)^
CAT117	*Ex* [ *myo-3p::venus* ]. (marker only)
CAT33	*Ex* [ *eft-3 p:: xdh-1* , *myo-3p::venus* ].
CAT118	*Ex* [ *eft-3 p:: xdh-1* , *myo-3p::venus* ].

(a) RB2379 was outcrossed to N2 five times in our lab before use.

(b) Outcrossed twice in National Bioresource Project (NBRP)-Japan.
